# “Courtesy stigma”: associative stigma experienced by Indonesian mental health nurses working in a psychiatric setting: a qualitative study

**DOI:** 10.1080/17482631.2026.2646245

**Published:** 2026-03-25

**Authors:** Muhammad Arsyad Subu, Henny Suzana Mediani, Janisha Kavumpurath, Alounoud Mohamed Salman Al Marzouqi, Jacqueline Maria Dias, Mini Sara Abraham, Vidya Seshan, Heba Khalil, Sawsan Abuhammad, Heba Hijazi, Fatma Refaat Ahmed, Nabeel Al-Yateem

**Affiliations:** a Department of Nursing, College of Health Sciences, University of Sharjah, Sharjah, United Arab Emirates; b Faculty of Nursing and Midwifery Universitas Binawan, Jakarta, Indonesia; c Faculty of Nursing Universitas Padjadjaran, Bandung, Indonesia; d Department of Health Care Management, College of Health Sciences, University of Sharjah, Sharjah, United Arab Emirates; e Faculty of Medicine and Health Sciences, University of East Anglia, Norwich, UK; f Department of Maternal and Child Health, Faculty of Nursing, Jordan University of Science and Technology, Irbid, Jordan; g Department of Health Management and Policy, Faculty of Medicine, Jordan University of Science and Technology, Irbid, Jordan

**Keywords:** Courtesy, association, stigma, mental illness, nurses, mental hospital

## Abstract

**Background:**

Mental health is crucial for emotional and personal well-being, healthy relationships, and effective community contribution. The stigma associated with mental illness often diminishes the therapeutic alliances between those with mental illness and healthcare practitioners. Courtesy stigma refers to the social disapproval people encounter due to their association with individuals from a stigmatized group. This study aimed to explore the associative stigma experienced by psychiatric nurses working with individuals with mental illnesses in an Indonesian psychiatric setting.

**Methods:**

This is a qualitative descriptive approach. We recruited 25 nurses from a psychiatric hospital in Makassar, Indonesia. We conducted semi-structured interviews, and the data were analyzed using a thematic analysis approach.

**Results:**

We identified four themes: 1) perspectives of working in a psychiatric hospital; 2) barriers to working in the psychiatric hospital; 3) elements of courtesy stigma; and 4) the reasons for courtesy stigma.

**Conclusions:**

This study found that nurses working in mental health hospitals experienced stigma by association with patients with mental illness. Public education and the expansion of community mental healthcare are essential measures to reduce the effects of associative stigma. Further research is necessary to identify the factors contributing to the preponderance of associations among mental health nurses employed in Indonesian mental health settings.

## Introduction

Mental health is an integral part of holistic well-being. Our overall well-being depends on our mental health, which has intrinsic and external significance. According to the World Health Organization (WHO, 2022), stress and vulnerabilities at the individual, societal, and structural levels interact in complex ways to shape mental health. Mental health encompasses the capacity to engage in productive daily activities (e.g., providing care), maintain healthy interpersonal connections, and effectively manage challenges and changes (Gaolaolwe et al., 2023; Njoku, 2022). Mental health is vital to emotional and personal well-being, to maintaining healthy relationships, and to contributing to one’s community or society.

Stigma is “an attribute that is deeply discrediting. It diminishes an individual’s social status by reducing them in the eyes of others from being whole and usual to tainted and discounted” (Goffman, 1963, p. 3). In general, stigma around mental illness has persisted for generations despite Erving Goffman originating the term ‘stigma’ in the 1960s (Yin et al., 2020). According to Stutterheim & Ratcliffe, (2021), stigma is a social phenomenon in which people perceived by the public as having inferior traits are subjected to unfair and unpleasant treatment. The stigma around mental illness has always persisted and has been present in literature from the Middle Ages onward (Yin et al., 2020). Stigmatization refers to a public perception of an individual as undesirable or atypical due to a socially discrediting characteristic or conduct (Mohammadzadeh et al., 2020). Engaging in that behavior causes significant distress among individuals repeatedly confronted with its consequences (Link & Stuart, 2017). Stereotypes originate from negative attitudes that lead to stigmatization (Avcil et al., 2016; Çaynak et al., 2021). How society applies labels determines how to distinguish between ‘us,’ which refers to the majority, and ‘them,’ which refers to the minority, who share the same negative or objectionable traits (Almuzini et al., 2020). An assumption of immediate danger is the most prevalent form of stigma directed at people with mental illnesses (Subu et al., 2022). A large number of people suffering from mental illness refrain from seeking treatment for their condition out of worries regarding potential harm to their family’s reputation, diminished marriage opportunities, prejudice, exclusion from society, and stigma (WHO, 2019). The stigma associated with mental illness impacts families whose members have been diagnosed, and it lowers their quality of life (Gaolaolwe et al., 2023).

“Stigma by association, also referred to as courtesy stigma, involves public disapproval evoked as a consequence of associating with stigmatized persons” (Phillips & Benoit, 2013, p. 139), and it is frequently focused on and redirected toward those close to those with mental illness (Phillips & Benoit, 2013). Undoubtedly, mental health nurses (MHNs) encounter stigma by association, not only from the broader populace but also from their peers in the nursing profession. This courtesy stigma may lead to isolation, negatively impact mental health, and possibly influence the quality of care provided by these individuals (Jones et al., 2024). In addition, stigma-by-association results in the erroneous perception that mental disorders are contagious, leading non-mental health professionals to believe that mental health nurses (MHNs) who treat patients with mental disorders are more likely to develop such disorders themselves (Yanos et al., 2017).

### Study purpose

The stigma surrounding mental illness continues to be a major barrier to psychiatric care in many countries. For example, this stigma affects not only individuals with mental health conditions but also their families and mental health workers in Ghana, Africa (Mensah, 2024). Similarly, mental health professionals, including nurses in Singapore, also experience this courtesy stigma (Picco et al., 2019). A study found that higher levels of associative or courtesy stigma were associated with greater burnout and lower job satisfaction (Yanos et al., 2020). The theoretical framing of courtesy stigma posits that stigma is a highly discrediting attribute that transforms how a person is perceived, shifting them from a complete, typical individual to someone viewed as tainted and marginalized. Courtesy stigma extends this concept by indicating that people near the primary stigmatized individual also bear some of this discredit, essentially undergoing 'stigma by association.' In addition, association value describes how people logically evaluate the importance of future interactions with someone. If associating with a person who has a low “Association Value”—for instance, because they are connected to a stigmatized individual—lowers one's social reputation, others might distance themselves, which could lead to social isolation for that person. Our thorough literature review revealed a lack of studies examining the stigma associated with MHNs in Indonesia. Research on the “courtesy stigma” among nurses in mental hospitals in Indonesia is crucial because this stigma is a construct that empirical researchers have relatively neglected. Additionally, it is important that ongoing efforts to investigate this underexplored yet important issue, combined with future initiatives to confront misconceptions about mental illness, are made. Therefore, it is essential to explore the prevalence of stigma by association among MHNs who provide care to patients with mental disorders in Indonesia. This study explored the associated stigma experienced by Indonesian nurses working in a psychiatric setting. An exploration of the consequences of associative stigma processes on mental health nurses could potentially contribute to a broader understanding of the intricate social dynamics inherent in mental health care.

## Methods

### Design

This study explored the courtesy stigma or associative stigma encountered by nurses who provide nursing care for people with mental illnesses in Indonesia. We adopt a descriptive qualitative method with thematic analysis (TA). The researchers adhered to the COREQ guidelines for qualitative studies (Tong et al., 2007). The COREQ (Consolidated Criteria for Reporting Qualitative Research) domains—(1) Research Team and Reflexivity, (2) Study Design, and (3) Analysis and Findings—serve to promote transparency and rigor in qualitative studies involving interviews or focus groups. In this study, COREQ domains influenced how interviewers were positioned, how data were collected, the analytical choices made, and the credibility of the findings. In qualitative research, the researcher acts as the primary instrument, although this influence is often not fully documented in final reports. It requires reflective thinking about how their background, experiences, and biases might affect data collection and analysis. For example, a nurse interviewer’s background helped shape how they interpreted participants’ stories, offering deeper insight into workplace issues and healthcare stigma. In line with the reflexivity principles in the Consolidated Criteria for Reporting Qualitative Research (COREQ), the researcher consistently assessed how their experiences and assumptions could influence their work. Qualitative research investigates individuals’ interpretations and meanings of social phenomena within specific contexts (Grossoehme, 2014). Qualitative epistemology investigates how knowledge is created, interpreted, and understood, focusing on subjective, context-specific, and socially constructed meanings rather than objective, universal truths. Its primary aim is to understand how individuals perceive and assign importance to their experiences (Herber et al., 2025). Unlike positivism, which seeks objective facts, qualitative epistemology regards reality as subjective, shaped by interactions, experiences, and interpretations. It aims to explore the processes behind human behavior, particularly how people interpret their worlds and find meaning (Herber et al., 2025). It is a method that aims to uncover themes, patterns, and meanings, and was employed to examine psychiatric nurses’ experiences in the context of mental illness (Braun & Clarke, 2006).

### Setting and participants

This study was conducted at a Hospital in Makassar, South Sulawesi Province, Indonesia. This government-run mental health facility offers a range of services, including psychiatric care, treatment for substance addiction, and additional psychological therapies. Twenty-five nurses (15 females and 10 males) were recruited purposively (purposive sampling) for the study. According to Charmaz (2006), 25 participants are sufficient for achieving data saturation in qualitative research. We consider the maximum variation in participant selection. Participants were approached face-to-face and via email. They ranged in age from 22 to 48 and held bachelor’s degrees in nursing (BN) with a concentration in psychiatric nursing. Participants must have at least 2 years of experience in the field. Each nurse participant was given a comprehensive explanation of the research’s objectives before the interviews and was made fully aware that their participation was entirely voluntary. Other factors considered when selecting participants were years of nursing experience in mental health settings. In this study, there were no direct relations between the authors and the participants. Each participant received a research information document containing an extensive overview of the study. Furthermore, they were informed that they could withdraw from the study at any time. Voluntary participation was provided without remuneration. In this study, none of the participants refused to participate or dropped out.

### Data collection

In this study, semi-structured interviews were the main data collection method. Interviews were conducted by four authors (MAS, HSM, US, and PN), all PhD holders and experienced in qualitative interviewing. They are faculty members in nursing at different universities in Indonesia. The interviewer’s professional training or practical experience can color their understanding and interpretation. For instance, a nurse researcher discussing mental health stigma with nurses may interpret responses through a healthcare lens, highlighting aspects such as patient care, workplace culture, and ethical considerations. Interviewer positioning requires reporting the interviewer’s credentials and experience. This encourages researchers to consider how their background—such as being a clinician-researcher—may affect participants’ responses or their interpretation of the data. Data generation procedures specify the sampling criteria, sample size, and data collection setting. They also emphasize elements such as using an interview guide, taking field notes, and reaching data saturation to ensure the data is thorough. Before formulating interview questions, a review of relevant literature and previous studies on mental health stigma was conducted.

Before the interview, every participant was required to read, sign a consent form, and furnish information about the research activity. The interviews lasted 25 to 45 minutes and were conducted using pre-established interview questions. To mitigate associative stigma among the participants, they could reflect on and scrutinize their responses throughout the interviews. All interview data were collected via audio recording at the participants’ workplace in the hospital. In this study, data saturation was achieved in interview 23. However, two remaining interviews were also conducted.

During the interview data collection, the interviewers asked participants a series of questions to gather information about the courtesy stigma they experienced. For example, we asked participants to provide a brief overview of their personal background, including their name, age, education, and occupation. We also asked about their opinions, feelings, or experiences related to working with mentally ill patients. Additionally, we asked about any experiences of discrimination and their feelings resulting from working with mentally ill patients. We questioned participants about negative feelings or experiences with others caused by working with mentally ill patients. Furthermore, we asked whether courtesy stigma affected their personal well-being or professional life. We asked if they had considered changing jobs or leaving the nursing profession due to stigma experiences related to working with mentally ill patients. We also asked whether they had received support from colleagues, leaders, or family. Participants were asked about the support they received because of stigma related to their work with mentally ill patients. In addition, we explored their coping mechanisms in response to courtesy stigma experienced while working with mentally ill patients. Finally, we asked if there was anything else participants would like to share about their experiences that had not been addressed during the interview.

**Semi-structured interview questions**

1. Can you tell me a little bit about yourself?
2. Have you ever felt negative feelings, been shamed by nurse colleagues, family, or the public due to your association with mentally ill patients?
3. Can you share your feelings about being treated differently due to the patients you care for or the nature of your work?
4. Can you share a time when you felt undervalued or treated unfairly because of your role as a psychiatric or mental health nurse?
5. Can you share whether your interactions with family, friends, or the general public have changed since you started working in this mental health specialty?
6. Who is aware of the details of your work, and how comfortable are you with them knowing?
7. Have you ever considered concealing your profession or the specific patient population you work with to avoid negative reactions?
8. Do you talk to anyone about these experiences of stigma? If so, who, and what do you discuss?
9. In what ways has experiencing courtesy stigma influenced your personal well-being or your professional life?
10. Has this experience ever made you consider changing your job or leaving the nursing profession?
11. In your opinion, do you feel that this associated stigma has impacted your job satisfaction?
12. Have you received any support from your colleagues, leaders, or family? If so, what kind?
13. What strategies have you used to cope with the negative opinions or behaviors of others?
14. Is there anything else you’d like to tell us about your experiences that we haven’t addressed?

### Data analysis

We conducted our thematic analysis according to the guidelines of Braun and Clarke (2006). Data analyses were conducted by four authors (MAS, HSM, and US). Additionally, all authors reviewed all the analyses and agreed. In this study, the authors read and reread all text responses to find the participants’ meaningful contributions. Manual coding was employed to process each text response. Following the responses, the researchers categorized the statements. Then, researchers focused on identifying issues, similarities, and differences that emerged from participants’ responses during the coding process. Furthermore, the researchers convened a meeting to reflect on the data as they conducted the analysis. It was determined that the codes assigned by each researcher accurately reflected the participants’ responses. Research team members agreed on the preliminary codes or identifiers during a meeting. Subsequently, the researchers revisited the responses and classified the significant statements they had discerned as conforming to the codes. Over several months of weekly meetings, the team deliberated significant statements and collectively revised or enhanced the code. The themes emerged by organizing the codes according to the participants’ quotations. The researchers developed the themes through careful, extensive use of the codes, staying true to the data. Multiple research team meetings, along with ongoing interactions and collaborations among researchers during the study, helped ensure the study’s credibility and rigor. In this study, all themes and subthemes were verified by every author. However, because the responses were anonymous, verification of members was not possible. In addition, study participants did not provide feedback on the research findings.

In our analysis process, we follow a qualitative coding approach—moving from raw, unorganized data to the development of themes—based on the interview study on “courtesy stigma.” Raw text data are the original and unedited transcript. It is the original information collected directly from an interview. A code (label) is a brief, word-based description of the main part of the data segment. A category (grouping codes) is a step in grouping similar codes, helping organize the data into manageable units. Lastly, the theme is a broad, interpretive phrase that explains 1) perspectives of working in a psychiatric hospital, 2) barriers to working in the psychiatric hospital, 3) elements of courtesy stigma, and 4) the reason for courtesy stigma.

#### Study rigor

The rigor standards for qualitative data proposed by Chiovitti & Piran, (2003) were followed in the current study. Elements comprising credibility, audibility, and suitability constituted this rigor criterion. Credibility is established through precise depictions or interpretations of a human phenomenon, enabling readers or individuals who have directly experienced it to connect with it. To build credibility, we revisited the participants to review transcripts or findings, gather feedback, and incorporate direct quotations from participants to support interpretations and confirm alignment between the raw data and the final findings. Manual, word-by-word, and line-by-line coding analyses were implemented to maintain robust correlation with the data. In this study, we used NVivo software for data management. Auditability refers to the ability of other researchers to employ the techniques and results developed by a particular investigator. Auditability pertains to the degree to which an additional researcher can discern the methodologies employed by the first and reach an indistinguishable conclusion. Chiovitti & Piran, (2003) define fittingness as the likelihood that the findings from a specific study can be extrapolated to analogous circumstances within a broader framework. An external researcher conducted an additional review of each component of coded data throughout the coding procedure.

#### Ethical consideration

The principles of the 1975 revision of the Declaration of Helsinki serve as the foundation for this research. The Ethics Committee of the University of Binawan in Jakarta, Indonesia, approved this research (No. 47/EP/KEP/UBINAWAN/IX/2024). Written informed consent was obtained after all nurses were provided with comprehensive oral and written information regarding the study. The MHNs were assured of confidentiality, guaranteeing that the findings would not reveal private information. The participants were duly informed of the voluntary nature of their participation, their right to withdraw from the research at any time, and that their decision to engage or abstain would not affect their capacity to participate in future studies. Before commencing data collection, we ensured that participants provided written informed consent and implemented stringent protocols to safeguard the confidentiality and anonymity of their information. To minimize the risk that transcriptions become associated with particular participants, each participant was assigned an alphanumeric designation (e.g., P1 represented Participant 1, P2 represented Participant 2, P3 represented Participant 3, and so on). Every interview recording was uploaded to a password-protected computer. The exclusive use of all interview materials, including transcriptions and recordings, is limited to the researchers.

## Results

The study participants consisted of 25 psychiatric nurses, 15 females and 10 males, aged 22 to 48 years. All nurses have a Bachelor of Nursing (BN) degree with a specialization in psychiatry. This study identified four distinct but connected themes that emerged from the interviews. These themes described the experiences of MHNs with courtesy stigma working with patients in a mental hospital: 1) perspectives of working in a psychiatric hospital, 2) barriers working in the psychiatry hospital, 3) elements of courtesy stigma, and 4) the reason for courtesy stigma.

### Theme 1: perspectives of working in a psychiatric hospital

Most participants expressed that employment at the hospital is essentially a privilege, distinguishing it from employment at other hospitals. A significant proportion of the participants expressed satisfaction with their workplace. They expressed the importance of assisting patients and demonstrated understanding of their obligations and professional conduct.

*I am happy to work at this hospital [psychiatry hospital]... Working at this hospital for me a better understand individuals with mental illness…A psychiatric hospital is a privileged institution. I can say that the approach to individuals here is unique. Helping patients makes me happy because they need help… I know of it...* (P4).

#### Subtheme: a right place to work

Some MHNs indicated that they had made the right choice to work in a mental hospital. They said that a mental hospital is the right place to work.

*Luckily, I am allowed to be employed here [hospital]. Regarding personal and social responsibility, I am convinced this is the ideal place to be and work. Being able to touch the lives of patients, particularly young patients, is of the utmost importance; therefore, I experience both happiness and sadness, but I adore my job.* (Participant 9).

#### Subtheme: relatives’ opinions about working in the mental hospital

Some MHNs expressed that family members and relatives had negative perceptions of hospitalized individuals with mental illness. There were worries regarding the patients’ propensity for violent behavior and the nursing staff’s potential for experiencing a deterioration in their mental health.

*......Relatives and friends consistently advise me about work there, suggesting that I seek employment elsewhere and questioning my decision not to transfer to another hospital; patients are assaulting me. Friends and family are highly concerned for us.* (Participant 19).

### Theme: barriers to working in the mental hospital

#### Subtheme: emotional and work overload

Some MHNs identified both physical and emotional overload as barriers. They reported that this hospital’s physical and emotional burden is significantly higher than that of other departments. When they encounter challenges at work, a lack of assistance causes them to encounter obstacles.

*… This hospital’s physical and emotional workload is significantly greater than other departments. I have held positions in various sectors, but the psychological and physical workload here is particularly high… We experience a physical and psychological low here...* (Participant 22).

#### Subtheme: difficult work

The nurses who participated in this study were asked about the challenges they faced within the healthcare facility.

*… yes, we have problems. In the beginning, I experienced difficulty due to the issues I encountered with patients’ diagnoses. Problems can arise when distinguishing between diagnoses due to their highly similar symptoms.* (Participant 24).

#### Subtheme: patients’ behavior

Some participants reported challenges associated with patients’ violent behaviors.

*Yes, it is stressful… Patient harassment is unavoidable. This could potentially manifest as both verbal and non-verbal harassment. During episodes of patients’ agitation, I encountered challenges in devising verbal methods to exert control and calm the patients…it is related to violent patient management.* (Participant 18).

### Theme: elements of associative or courtesy stigma

#### Subtheme: labeling

Because they work in psychiatric hospitals, nurses who are labeled crazy nurses frequently experience humiliation and insult.

*It is a label. I frequently hear or respond to the following insults from others...employed in a mental institution?... Working with the insane? Nonetheless, I assert that I am a mental health nurse and not a crazy nurse, even though they perceive me to be such an insane nurse. That is a label. One says, “Whew, a psychiatric nurse”? They continue to label nurses…we have been labeled by our peers [nurses].* (Participant 10).

#### Subtheme: stereotyping

Associating a person with stigmatized populations, such as mentally ill patients, perpetuates preconceived notions about psychiatric mental health nurses. It is believed that the psychiatric institution is a hazardous work environment due to the violent and dangerous nature of the mentally ill, which nurse participants discussed. General nurses, in addition to community members, hold the belief that the mental health nurse faces physical and mental threats.

*… Yes, [the psychiatric hospital] is regarded as a harmful or unsafe work environment. Friends have informed me that they have, sir. Whew... you work in that hospital? Do you have employment at a mental hospital? They believe it must be difficult to work here. It is perceived as an area susceptible to assault. It is described as a location where individuals are forced to come rather than arriving voluntarily.* (Participant 16).

Additional participants indicated that their association with psychiatric patients contributed to the general public’s perception of them as mentally ill.

*… care for the mentally ill causes them to treat you as if you were a mental patient. It is commonly believed that after an extended period of service in this field, one begins to exhibit patient-like behavior. That would tarnish your image if you remained in this job for a long time.* (Participant 1).

#### Subtheme: a non-prestigious work

The nature of MHNs’ work continues to baffle other nurses and the general public. The general population and non-psychiatric nurses believe that working in a mental hospital is not prestigious.

*… I believe that non-psychiatric nurses have a low regard for mental health nurses….“Why would you want to work in a mental hospital when there is nothing to do there? I favor treating mental health nursing as a more contemporary or modern discipline. We want others to share positive opinions about mental health nursing…* (Participant 22).

#### Subtheme: insulting and status loss

Because they care for hospitalized patients with mental illness, MHNs are subject to insults from outside the field of mental health nursing. Even her family insulted her as a nurse. A nurse indicated that her family members still insulted psychiatric nurses.

*They [family members] insult me… I clarified at a family gathering that mental illness does not constitute a contagious disease. Despite being my family members, they continue to reject it. When my sisters-in-law and my husband’s family discovered that I work at the hospital, they said, ‘Whew, do you work at the mental hospital?’ You pick up by your spouse, right? You can imagine. You agree that I was angry, correct?* (Participant 24).

#### Subtheme: discrimination

Discrimination was described by the mental health nurses (MHNs) regarding their workplace, family-related, and public. For example, study participants reported that the general public considered MHNs to be unqualified to practice nursing.

*Sadly, some individuals do not even acknowledge that we are nurses... Occasionally, members of the public fail to acknowledge our affiliation with the hospital. I believe that the public does not always regard us. They discriminate against us, right? We face discrimination in every situation. Indeed, they do receive these items regularly, and they receive their portion before we do. As you know, we face discrimination even in training… we are rarely included.* (Participant 14).

### Theme: the reason for courtesy stigma

Participants in the study identified courtesy stigma as the form of stigma they encountered during their ongoing interactions with patients. They stated that their affiliation with individuals living in mental hospitals who were afflicted with mental illness led to stigmatization. A male nurse said:

*… The general public holds the belief that one cannot adequately care for a mental patient if they do not suffer from a mental illness. Although it is occasionally put into jokes, the statement is quite distressing. Yes, no doubt about it…Yes, stigma. I believe that the stigmatization of our profession is because we work with mentally ill patients. We are subject to the same stigmatization as the patients under our care. They fail to know our emotions and understand our feelings.* (Participant 12).

## Discussion

This study explored the associated stigma experienced by Indonesian nurses working in a psychiatric setting. This study is essential because it explores the phenomenon of stigma by association in relation to nurses employed in mental health settings. It contributes to the body of knowledge regarding its effects on nurses and the nursing profession. Stigmatization may make it more difficult for people with mental illness to receive effective therapy and seek help (Mohammadzadeh et al., 2020; Zolezzi et al., 2018). The stigma by association that nurses in psychiatric settings experience has not received much attention in the current literature (Almuzini et al., 2020).

In this study, mental health nurses (MHNs) provide their perspectives on working in a psychiatric hospital. The working environment significantly impacts the mental health and overall well-being of MHNs who are employed. They emphasized the importance of providing help to patients and demonstrated a clear understanding of their responsibilities and professional attitudes. These nurses are generally satisfied with their jobs in the mental health field. Positive aspects of working in the mental health field include the concepts of contentment and well-being (Picco et al., 2017). This study also found that participants expressed their decision to work in a mental hospital as appropriate and that their experience was excellent. MHNs indicated that they had the right choice to work in a mental hospital, and a mental hospital is the right place to work. However, some participants claimed that their family members and relatives had an unfavorable opinion of people who were in mental hospitals. The belief that individuals with mental disorders pose a threat is the most prevalent form of stereotyping regarding patients with such conditions (Subu et al., 2021). A considerable proportion of individuals with mental illness withhold from seeking treatment due to concerns regarding potential negative repercussions on their family’s standing, reduced prospects for marriage, prejudice, social exclusion, and stigma (WHO, 2019).

Our study results indicated that there are some challenges or barriers that MHNs face when working in psychiatric hospitals. Similarly, Kuek et al., (2021) identified three barriers: an unclear role, hostility from non-peer staff, and an unsupportive work environment. The World Health Organization - WHO (2010) identified five key barriers: 1) the exclusion of mental health from the public health agenda and the funding implications; 2) the existing structure of mental health services; 3) the absence of integration within primary care; 4) insufficient human resources dedicated to mental health; and 5) the dearth of public mental health resources.

Our study findings also indicated that nurses are concerned about work overload in mental hospitals. Consistent with our findings, work overload negatively correlates with job satisfaction, as increased workload is associated with decreased job satisfaction (Marco et al., 2008). In mental health services, excessive workload or work overload negatively impacts employees’ well-being, health, and the standard of care delivered to clients. The utilization of personal attire by healthcare workers in psychiatric services has thus been the subject of recent scholarly studies. The nursing staff of private mental health facilities reported higher average workloads (Souza et al., 2015). Work can have a significant impact on one’s health and well-being. It can serve as a vital social outlet, bolster self-esteem, and impart a sense of identity (Robinson & Smith, 2021). One of the subthemes we identified is the challenging work in mental health facilities. Work-related difficulties that frequently adversely affect mental well-being include extended, rigid work schedules, personnel shortages resulting from downsizing or unfilled positions, and an increasingly burdensome workload. Work difficulties in mental health care contexts may also include a hostile work environment that promotes bullying, harassment, or abuse; inadequate training or direction regarding the responsibilities of employees; and restricted or ambiguous communication from management regarding tasks, objectives, or decision-making processes (Robinson & Smith, 2021).

Our study participants indicated that negative patient behavior is a barrier to working in psychiatric settings. More than fifty percent of the mental healthcare cohort reported having been physically assaulted or threatened (Liyanage et al., 2018), which is consistent with this result. The incidence of this form of maltreatment was most significant among mental health unit nurses. A shift in perspective toward zero tolerance for violence and promoting workplace harmony should be a priority (Almuzini et al., 2020). In this study, we also found some elements of courtesy stigma. This stigma refers to hostility directed toward an individual because of their association with a stigmatized group or person (Hamilton & Braithwaite, 2016). Despite making substantial contributions to the management of mental illness, MHNs continue to face stigmatization in the course of their professional endeavors (Buertey et al., 2020).

In this study, labeling is one of the elements of courtesy stigma. MHNs who are labeled ‘crazy nurses’ frequently experience humiliation and insults because they work in psychiatric hospitals. According to Hing et al., (2016), stereotypes, fear, embarrassment, anger, rejection, or avoidance are just a few examples of the negative emotions that frequently accompany the behavioral manifestation of stigma. Feeling uncomfortable with disclosing one’s work, attributing mental illness to mental health professionals, preconceived notions about the efficacy of mental health services, and preconceived notions about the discomfort of working with people who have severe mental disorders have been identified as key forms of associative stigma (Lin et al., 2019). Other studies revealed that MHNs were sometimes referred to as ‘crazy’, resulting in diminished self-esteem. Society considers mental health personnel to be insane, and psychiatry is regarded as a profession that diminishes an individual’s status in society (Ebsworth & Foster, 2017; Vayshenker et al., 2018).

Courtesy stigma is a phenomenon in which the stigma of the disease becomes associated with caregivers due to the interaction between MHNs and mental patients (Lin et al., 2019). Another element of courtesy stigma found in this study is stereotyping. Consistent with our recent findings, prior scholars have found that MHNs experience stereotypes and are undermined in the performance of their responsibilities (Ebsworth & Foster, 2017). Additionally, it has been noted that MHNs face judgment from individuals outside the domain of mental health nursing because they provide care for hospitalized patients with mental illness. Psychiatric nurses were stigmatized to the extent that they were deemed incapable of providing effective treatment to mentally ill patients and careless by nonpsychiatric nurses (Ben-Natan et al., 2015). A study conducted by Picco et al., (2019) found that a significant proportion of respondents refrain from discussing their work with individuals external to the mental health system and report frequently encountering negative stereotypes regarding mental health service recipients and mental health professionals. Additionally, this research indicated that individuals appeared concerned with nurses’ safety and well-being. The majority of individuals seeking mental health care were perceived as dangerous, pessimistic, immature, callous, harmful, and possessing inadequate personal sanitation (Hamdan-Mansour & Wardam, 2016). The safety of MHNs was regarded as the highest priority; consequently, a segment of the public with good intentions demanded that the government implement policies to ensure MHNs’ well-being and facilitate their work. Additionally, the results of this study align with prior research, which found that personnel providing medical care regard individuals with mental illness as more threatening, unpredictable, and demanding compared to those in other settings (Wang et al., 2018). These characteristics thereby increase the safety of nurses in the workplace. Family members and other medical professionals maintain concerns about mental health facilities and patients. Previous academics have supported the findings regarding the safety of MHNs (Thornicroft et al., 2016). Similarly, caring for individuals with mental illness in the workplace poses a danger, and several nurses have experienced dangerous situations in this role, including aggressive interactions with patients (Opare et al., 2016). We also found in this study that non-prestigious work in mental health settings is an element of courtesy stigma. Some of our participants expressed that their occupation is straightforward, necessitating only common sense, and potentially doable by any individual. Likewise, several participants mentioned that community members frequently declare they could never do that work (Wang et al., 2018).

Mental health professionals receive little recognition because they work within a low-status discipline compared to healthcare professionals who serve other patient populations (Hansson et al., 2014). According to our study findings, MHNs experience status loss due to insults from others. Other non-MHN colleagues viewed the MHNs in contempt because of their perception that they were considered mentally ill (Corrigan et al., 2014; Henderson et al., 2014). The majority of nurses working in mental health units were aware that they might feel inferior to their coworkers in other health fields when it comes to the humiliating situations they encounter in the workplace and every aspect of life as a result of stigma by association (Almuzini et al., 2020). Moreover, a study found that 50% of participants said they had heard derogatory remarks regarding the incompetence of mental health personnel or experienced such remarks at least twice (Catthoor et al., 2014). Additionally, we identified discrimination against MHNs as a key finding of this study. Discrimination, both within and outside of hospitals, was considered painful by MHNs. General nurses in the same health facility were treated much more with respect than mental health nurses (MHNs) (Opare et al., 2016). Mental illness patients and their healthcare providers are subject to discrimination (Crabb et al., 2012).

Courtesy stigma refers to a negative characterization that affects individuals due to their association with a stigmatized person or group. Courtesy stigma was extended to include those who merely associate with individuals who have become negatively branded. Stigmatization may result in adverse consequences, including reduced self-esteem, conflicts within family members, and implications for employment prospects (Picco et al., 2019; Subu et al., 2021). This study found some reasons for courtesy stigma or stigma by association. Stigma related to mental health nursing and psychiatry has reportedly diminished their standing in the eyes of the general public in the context of health care (Yanos et al., 2017). Several studies have documented examples of stigma directed at individuals with mental illnesses. However, stigma by association refers to the stigmatization that extends beyond the individuals themselves to include mental health.

professionals and family members (Picco et al., 2019; Ubaka et al., 2018). There exists a correlation between associative stigma directed at mental health professionals and diminished levels of job satisfaction. Consequently, this downward spiral may lead to substandard patient care, a potential exacerbation of the chronic nature of the disorders, and treatment discontinuation (Picco et al., 2019). Other research evidence indicates that stigma associated with mental disorders is present in every country worldwide (Rossler, 2016). In developed countries, the stigma associated with mental illness remains significant, with approximately 90% of individuals reporting experiencing stigmatization. However, developed countries possess a greater capacity for human resources to support mental health care (Henderson et al., 2020; Njaka, 2021). Medical and health science students in most developing countries do not consider psychiatry a viable academic choice. Most developing countries neglect and inadequately fund mental health services (Dauda, 2016). According to Stanley et al., (2023), to successfully deal with the associative stigma that mental health professionals face and the potential repercussions it may have on mental health services, it is imperative to establish policies, protocols, and educational initiatives. The aforementioned necessitates evidence from various sources to develop globally; thus, this is the first review on the courtesy stigma against mental health professionals worldwide.

Finally, our research advances understanding and expands the concept of courtesy stigma, offering insights that build on Erving Goffman's original idea (1963). Previous research on stigma mainly focused on family members of those with stigmatized conditions. However, recent findings indicate that healthcare providers also experience a form of courtesy stigma. This type of stigma involves negative perceptions, discrimination, or social disapproval directed at individuals connected to someone with a stigmatized identity, including nurses working with mental health patients. It is recognized as an issue in workplaces and professions, not just a family concern, and it also affects nurses. Such courtesy stigma can negatively impact job satisfaction, professional identity, and career growth among mental health nurses (Happell et al., 2014). Today, it’s seen as a complex, multi-dimensional social issue rather than an isolated personal experience. The study also highlights how courtesy stigma impacts nurses psychologically and professionally, especially in healthcare settings where nurses treating mental health patients often face inadequate support, discrimination, or negative attitudes from colleagues across departments. These experiences can increase workplace stress and hinder teamwork. Addressing stigma in healthcare is crucial to creating supportive environments for nurses.

### Study limitations

A limitation of this study was its small sample size, drawn from a single healthcare facility in Indonesia. While the qualitative insights offer detailed perspectives on specific experiences, they are not sufficiently representative to support generalization to larger populations or different settings. Caution is advised when applying these results broadly. Discussing stigmatization can be emotionally taxing for participants, which might affect their responses or cause distress during interviews. Nurses may be hesitant to fully disclose their experiences of courtesy stigma due to shame or a desire to project a positive image of themselves or their workplace, potentially impacting data accuracy. Additionally, recruiting nurses who have experienced courtesy stigma was difficult, possibly leading to a sample that does not encompass all experiences. Additional limitations include the potential normalization of stigma within institutions and the influence of interviewer positionality in psychiatric mental health systems, as well as the cultural specificity of Indonesian psychiatric nursing. Institutional normalization of stigma is the incorporation of systemic biases into organizational policies, procedures, and cultural norms, leading to the marginalization of stigmatized groups. In mental healthcare, the language used by healthcare professionals and existing power dynamics can sustain stigma, particularly against groups with migration backgrounds, by framing them negatively. Additionally, cultural narratives and media often associate mental illness with violence or sensationalize these conditions, reinforcing public stigma and influencing attitudes and behaviors in workplaces and schools. Another limitation involves the interviewer's role within Indonesia's psychiatric system and the cultural context of Indonesian mental health nursing. Interviewers working within these institutions may introduce bias and power imbalances, compromising objectivity and making it harder for patients to disclose openly. Moreover, the institutional setting can assign specific roles to patients, thereby objectifying their experiences and reducing the depth of interaction.

## Conclusion

Stigmatization may result in adverse consequences, including reduced self-esteem, family conflicts, and implications for employment opportunities. A large number of mental health nurses (MHNs) acknowledged experiencing stigmatization when discriminated against based on their profession or when others perceived their conduct to be identical to that of their patients. The current study concluded that nurses who provide care for mentally ill patients are stigmatized as a group. MHNs were stigmatized, labeled, and discriminated against, and their reputation was poor. Concerns were also expressed regarding the safety and well-being of nurses, even though they were subject to associative stigma. Nurses, on the other hand, provide clients with mental healthcare at their doorstep, and their contributions and value should not be underestimated. As a result, hospitals need to limit the dissemination of negative attitudes toward them. The present study indicates that stigmatized situations involving nurses working in mental health settings do occur, according to the findings of the present study. To lessen the stigma associated with mental illness, mental health nurses have also made recommendations. Additionally, a statistically significant distinction was observed in their responses when they heard others describe their work as simple or accessible to anyone. Furthermore, stigmatization is a prevalent issue among nurses who are employed in mental health settings in Indonesia. Additional study is warranted to explore healthcare providers' capacity to dismantle stigma by association through education and to prepare novice nurses for practical experiences in mental health nursing. In addition, attempts were made to collect a more inclusive sample of MHNs employed in various areas of Indonesia to determine whether the prevalence of associative stigma encountered by MHNs in this research is representative of the profession nationwide.

### Implications for nursing practice

Our study findings have highlighted concerns that have implications for the professional responsibilities of MHNs. Nurses have acknowledged that when they are subjected to discrimination based on their profession, they experience stigmatization, which deters them from pursuing a career in mental health nursing. A significant proportion of MHNs believed that their profession elicited derision from others and that their patients' conduct influenced their own behavior. Their self-esteem may be negatively impacted, and they may withdraw from other healthcare providers as a result of courtesy stigma. Stigma reduction efforts should be directed toward MHNs. In light of the results presented here, policymakers and practitioners in mental health nursing should address the obstacles posed by stigmatization. Stigma campaigns ought to prioritize the education of individuals who have experienced mental illness to assist them in rejecting self-stigmatization and effectively confronting or circumventing public stigma. This may contribute to improved efficacy of mental health services in Indonesia.

## Data Availability

De-identified data and the study instruments will be made available by the corresponding author upon reasonable request for research purposes, subject to institutional approvals.
